# Multi-Aspect Optoacoustic Imaging of Breast Tumors under Chemotherapy with Exogenous and Endogenous Contrasts: Focus on Apoptosis and Hypoxia

**DOI:** 10.3390/biomedicines9111696

**Published:** 2021-11-16

**Authors:** Angelos Karlas, Antonio Nunes, Wouter Driessen, Evangelos Liapis, Josefine Reber

**Affiliations:** 1 Chair of Biological Imaging, School of Medicine, Technical University of Munich, 81675 Munich, Germany; angelos.karlas@tum.de (A.K.); antonio.nunes@bluepharmagroup.com (A.N.); evagelos2005@hotmail.com (E.L.); 2 Institute of Biological and Medical Imaging, Helmholtz Zentrum München (GmbH), 85764 Neuherberg, Germany; 3 Clinic of Vascular and Endovascular Surgery, Klinikum Rechts der Isar, 81675 Munich, Germany; 4 German Centre for Cardiovascular Research (DZHK), Partner Site Munich Heart Alliance, 80636 Munich, Germany; 5 Bluepharma-Indústria Farmacêutica SA, S. Martinho do Bispo, 3045-016 Coimbra, Portugal; 6 iThera-Medical GmbH, 81379 Munich, Germany; wouter.driessen@roche.com; 7 Pharmaceutical Sciences, Pharma Research and Early Development (pRED), Roche Innovation Center Basel, F. Hoffmann-La Roche Ltd., 4070 Basel, Switzerland

**Keywords:** photoacoustics, multispectral optoacoustic tomography, MSOT, breast cancer, molecular imaging, contrast agent

## Abstract

Breast cancer is a complex tumor type involving many biological processes. Most chemotherapeutic agents exert their antitumoral effects by rapid induction of apoptosis. Another main feature of breast cancer is hypoxia, which may drive malignant progression and confer resistance to various forms of therapy. Thus, multi-aspect imaging of both tumor apoptosis and oxygenation in vivo would be of enormous value for the effective evaluation of therapy response. Herein, we demonstrate the capability of a hybrid imaging modality known as multispectral optoacoustic tomography (MSOT) to provide high-resolution, simultaneous imaging of tumor apoptosis and oxygenation, based on both the exogenous contrast of an apoptosis-targeting dye and the endogenous contrast of hemoglobin. MSOT imaging was applied on mice bearing orthotopic 4T1 breast tumors before and following treatment with doxorubicin. Apoptosis was monitored over time by imaging the distribution of xPLORE-APOFL750©, a highly sensitive poly-caspase binding apoptotic probe, within the tumors. Oxygenation was monitored by tracking the distribution of oxy- and deoxygenated hemoglobin within the same tumor areas. Doxorubicin treatment induced an increase in apoptosis-depending optoacoustic signal of xPLORE-APOFL750© at 24 h after treatment. Furthermore, our results showed spatial correspondence between xPLORE-APO750© and deoxygenated hemoglobin. In vivo apoptotic status of the tumor tissue was independently verified by ex vivo fluorescence analysis. Overall, our results provide a rationale for the use of MSOT as an effective tool for simultaneously investigating various aspects of tumor pathophysiology and potential effects of therapeutic regimes based on both endogenous and exogenous molecular contrasts.

## 1. Introduction

Breast cancer is the most common solid tumor in women, with a pathophysiology involving multiple biological/molecular processes, such as cellular proliferation, metastasis, angiogenesis, inflammation, impairment of the immune response, hypoxia and apoptosis [1,2,3,4,5,6]. Simultaneous monitoring of multiple such processes would enable a multi-aspect disease characterization, leading to deeper understanding of its pathophysiology or improved diagnostics.

Imaging can enable the simultaneous monitoring of multiple biological/molecular processes involved in breast cancer by combining, for example, different contrast mechanisms in a single scanning session. This requires the use of hybrid (e.g., positron emission tomography-computed tomography/PET-CT or positron emission tomography-magnetic resonance imaging/PET-MRI, and fluorescence molecular tomography-computed tomography/FMT-CT [7,8,9]) or advanced stand-alone techniques (e.g., spectral CT and multiple-contrast MRI [10,11]). Nevertheless, the above-mentioned techniques might necessitate the synchronous administration of several contrast agents [12,13], a strategy with limitations, such as the increased possibility to trigger adverse effects (e.g., allergic reactions and renal failure). Furthermore, such hybrid/advanced modalities require highly specialized personnel and expensive and bulky equipment: features that can hamper their disseminated use or might increase patient inconvenience (e.g., due to claustrophobia) [14]. Also, many of these techniques employ ionizing radiation or suffer from poor spatial resolution (e.g., CT and PET). Exemplary techniques that can overcome some of these limitations are fluorescence imaging or medical hyperspectral imaging (HSI) [15,16], that, however, might suffer from poor penetration depth. Thus, novel stand-alone, non-ionizing and highly portable imaging technologies that can support the simultaneous imaging of different biological/molecular processes implicated in breast cancer by employing as few contrast agents as possible, is a valuable alternative.

Multispectral optoacoustic tomography (MSOT) is such a technique that can discriminate between different molecular chromophores purely based on their spectral signatures [17,18]. The optoacoustic contrast arises when light pulses are absorbed by the chromophores and ultrasound waves are produced in response. The chromophores can be endogenous, such as oxygenated (HbO_2_) and deoxygenated (Hb) hemoglobin, lipids and water, or exogenously administered agents, such as dyes and nanoparticles [17,19]. Based on this principle, MSOT has already been used in several preclinical, translational or even clinical studies, such as: (i) label-free imaging of tissue perfusion/oxygenation in breast cancer, inflammatory bowel diseases, cardiovascular and endocrinology applications, the musculoskeletal system and adipose tissues [17,20,21,22,23,24,25,26,27,28,29,30] or (ii) imaging of genetically-encoded probes, small-molecule dyes, nanoparticles (e.g., gold and carbon) or photoconvertible proteins [19,31,32,33].

Herein, we employed the capability of MSOT to resolve both endogenous and exogenous contrasts by imaging two fundamental aspects of breast cancer pathophysiology: apoptosis and hypoxia. We conducted targeted experiments in mice with breast tumors under doxorubicin chemotherapy and imaged tumor oxygenation based purely on endogenous hemoglobin contrast while in parallel monitoring apoptosis by visualizing the distribution of an injected poly-caspase-targeting agent (xPLORE-Apo-FL750©). Our results demonstrate the great capability of MSOT, a novel molecular non-ionizing and highly-portable technology, to simultaneously monitor different aspects of tumor pathophysiology based on both exogenous and endogenous contrast agents. The study is geared towards preclinical models nevertheless with great implications for translational research and clinically oriented studies or even improvement of future breast cancer diagnostics.

## 2. Methods

### 2.1. In Vitro Characterization of Exogenous Contrast Agents

To image apoptosis we employed the near-infrared (NIR)-dye-labelled probe xPLORE Apo FL750©. The probe was selected because it was labelled with an NIR-dye, taking into account that the laser of the MSOT system illuminates at the NIR region of the light spectrum (680–960 nm). The employed probe is a peptide-based polycaspase-binding-inhibitor probe (Val-Ala-Asp (OMe)) which specifically binds to the active center of the caspases 1, −3, −4, −5, −6, −7, −8 and −9: significant mediators and frequent targets for molecular imaging probes of apoptosis [34]. xPLORE Apo FL750© is the active compound binding to caspases whereby xPLORE-CTRL-FL690© is a non-specific compound which serves as internal control. To explore the performance of xPLORE-Apo-FL750© and xPLORE-CTRL-FL690© as exogenous optoacoustic contrast agents, we designed a series of in vitro experiments.

**Figure 1:**
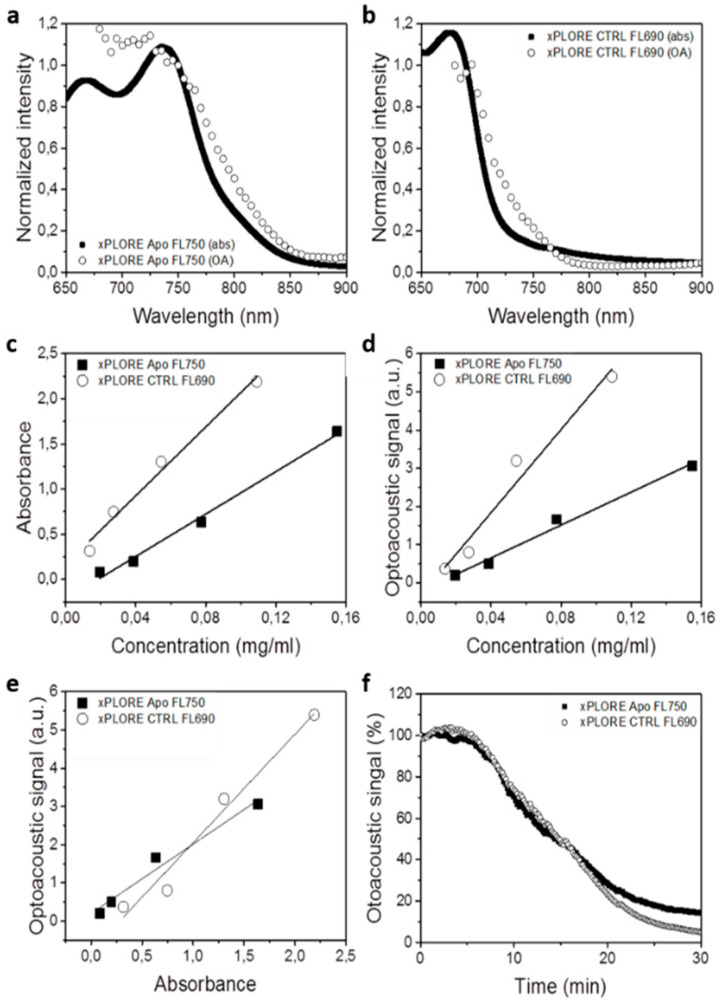
In vitro performance of xPLORE-Apo-FL750© and xPLORE-CTRL-FL690© as optoacoustic contrast agents. Normalized optical absorbance (abs) and normalized optoacoustic (OA) signal of (**a**) xPLORE-Apo-FL750© and (**b**) xPLORE-CTRL-FL690© at 650–900 nm light wavelength range. (**c**) Correlation between concentration and optical absorbance for each probe. (**d**) Correlation between concentration and optoacoustic signal for each probe. For all measurements shown in panels (**c**,**d**), the probes were superficially injected in agar phantoms and illuminated at 700 nm. (**e**) Optoacoustic signal for each probe as a function of optical absorbance. (**f**) Photobleaching of each probe over time.

First, we measured the optical absorbance of both probes (Figure 1). For this purpose, four saline dilutions of xPLORE-APO750© and xPLORE-CTRL-FL690© were prepared with optical densities ranging from 0.1 to 2.2 (0.1, 0.8, 1.5, 2.2), which corresponded to probe concentrations ranging from 0.02 to 0.16 mg/mL. Next, the absorption spectrum of each dilution was measured at the range of 650–900 nm with a spectrophotometer (USB4000, Ocean Optics, Florida, FL, USA). The dilutions were then sequentially injected and measured in a tissue-mimicking cylindrical phantom of 2 cm in diameter, which was prepared as described elsewhere [35]. In brief, the phantom consisted of 1.3% agar (Sigma-Aldrich, St Louis, MO, USA) for jellification and a 20% intralipid emulsion (Sigma-Aldrich, St. Louis, MO, USA) for light diffusion. A cylindrical inclusion of 3 mm in diameter was positioned in the middle of the phantom and was employed for containing the probe dilutions. A tube containing India ink with an absorbance of 0.3 OD at 800 nm was also placed in parallel to the abovementioned cylindrical inclusion for internal signal intensity reference.

### 2.2. Animal Handling

**Figure 2:**
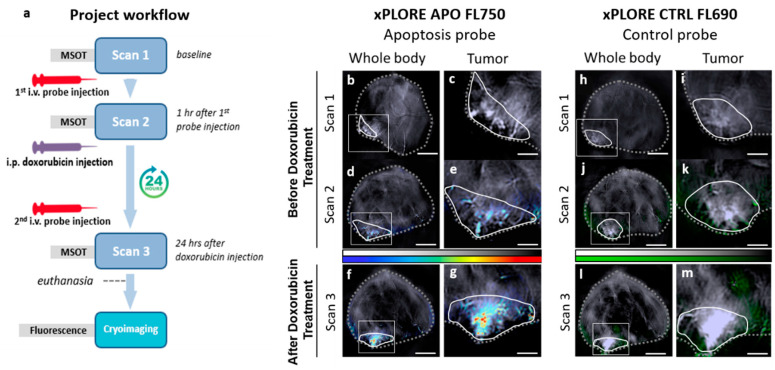
MSOT imaging of xPLORE-Apo-FL750© for the assessment of tumor apoptotic responses to doxorubicin therapy. (**a**) Schematic diagram summarizing the therapeutic, MSOT imaging, and cryoimaging schemes. (**b**–**m**) Transverse anatomical whole body MSOT images of mice bearing 4T1 tumors were collected at 800 nm and are shown in grayscale. These anatomical images were overlaid with the signal induced by xPLORE-Apo-FL750© (jet scale) or xPLORE-CTRL-FL690© (green scale). Animals were imaged with MSOT prior to administration of xPLORE probes (**b**,**c**,**h**,**i**—Scan 1), then injected with probe solution mixture and imaged again 1 h later (**d**,**e**,**j**,**k**—Scan 2). Next, mice were treated with doxorubicin for 23 h, re-injected with the probes and imaged again 1 h later using MSOT (**f**,**g**,**l**,**m**—Scan 3). (**b**–**g**) Results obtained for xPLORE-Apo-FL750©. (**h**–**m**) Results obtained for xPLORE-CTRL-FL690©. Insets (**c**,**e**,**g**,**i**,**k**,**m**) shown next to whole animal (**b**,**d**,**f**,**h**,**j**,**l**). MSOT images are magnified views of the corresponding tumor regions of interest.

Animal experiments were approved by the government of Upper Bavaria. Female nude mice (*n* = 6, 8–10 weeks old Nude-1 Foxn1; Harlan Laboratories, Eystrup, Germany) were inoculated orthotopically in the inguinal mammary fat pad with 1 × 10^6^ 4T1 mouse mammary adenocarcinoma cells (ATCC-CRL-2539, #5068892) suspended in a total injection volume of 50 µL. At the onset of therapy on day #1, the average tumor volume was ≈100 mm^3^. The workflow of the MSOT imaging study is outlined in Figure 2a. Multiple in vivo MSOT imaging sessions were performed at different time points: (i) on day #1, before injection of the probes (Scan 1), (ii) on day #1, 1 h after the intravenous administration of 200 µL xPLORE© injection mixture consisting of xPLORE-Apo-FL750© solution (100 µL) and xPLORE-CTRL-FL690© solution (100 µL) (Scan 2) and (iii) on day #2, 23 h after the intraperitoneal administration of doxorubicin treatment (Scan 3). The xPLORE injection mixture was prepared according to manufacturer’s instructions (xPLORE© apoptosis detection kit, iThera-Medical GmbH, Munich, Germany) and injected via the tail vein using a catheter. Another group of animals (*n* = 6) were injected intravenously with 0.9% NaCl (control group). Immediately after MSOT Scan 2, mice were injected intraperitoneally with 10 mg/kg doxorubicin (Sigma Aldrich, Taufkirchen, Germany). Finally, after the last MSOT imaging session (Scan 3), the animals were sacrificed and frozen to −80 °C for ex vivo fluorescence cryoimaging.

### 2.3. MSOT Setup, In Vivo Measurements and Data Analysis

Optoacoustic measurements were performed using a preclinical MSOT imaging system dedicated for small animal imaging (inVision 256-TF, iThera-Medical GmbH, Munich, Germany), already described elsewhere [36]. Optical excitation was provided by a Q-switching Nd: YAG laser with a pulse duration of 10 ns at a repetition rate of 10 Hz and a tunable range of 680–960 nm. Light was delivered to the sample chamber using a fiber bundle split into 10 output arms. The ultrasound signal was detected using a 256-element transducer array cylindrically focused with a central frequency of 5 MHz for transverse plane imaging.

Mice were anesthetized using a concentration of 1.8% isoflurane in oxygen and placed in the MSOT sample holder as described previously [37]. In brief, animals were placed onto a thin clear polyethylene membrane in a holder and positioned in the water bath maintained at a temperature of ≈34 °C for optimal acoustic coupling. Each scan lasted for ≈20 min and multiple cross-sectional images throughout the entire mouse volume at the level of the tumor were acquired. Data acquisition was performed by averaging 15 frames per wavelength at 690 nm, 700 nm, 715 nm, 730 nm, 750 nm, 760 nm, 800 nm, 825 nm, 850 nm, and 900 nm (10 wavelengths in total). Finally, recorded multi-wavelength MSOT data were spectrally unmixed for each probe based on their specific optoacoustic spectra. The average optoacoustic signal intensity was quantified by region-of-interest (ROI) analysis of the recorded MSOT images corresponding to the time points before and after the treatment with doxorubicin. For each scan, a ROI including the entire tumor area was manually delineated by an expert with long experience in preclinical MSOT tumor imaging. The same scaling was used for all images within the series. Manual segmentation of the ROIs was performed in MATLAB© software (v. 2019b, Mathworks™, Natrick, MA, USA). We used Origin© software (v. 9.3, OriginLab™, Northampton, MA, USA) for the creation of the graphs and R software (v. 3.6.3, R Core Team, Vienna, Austria) for the performed statistical analysis (calculation of the effect size and confidence intervals). The error bars presented throughout the current work correspond to standard error of the mean.

### 2.4. Ex Vivo Fluorescence Cryoimaging

To further validate the presence of the injected xPLORE apoptosis probe into the tumor, we performed ex vivo fluorescence cryoimaging of the scanned animals. The sacrificed animals were embedded in optimal cutting temperature compound (Tissue-Tek, Zoeterwonde, The Netherlands) and cryosliced along the axial planes at steps of 100 µm using a cryotome (CM 1950; Leica Microsystems, Wetzlar, Germany). The cryotome was equipped with a CCD camera to capture fluorescence signal from the surface of the remaining bulk mouse tissue sample after each slicing step and thus visualize the 3D-distribution of the fluorophore within the whole sliced volume [38].

## 3. Results

Assessment of injected probes as optoacoustic contrast agents in vitro.

We first conducted a series of in vitro experiments to assess the performance of the following probes as optoacoustic contrast agents: the apoptosis-targeting, poly-caspase binding agent (xPLORE-Apo-FL750©) and a control agent unable to bind caspases (xPLORE-CTRL-FL690©). Figure 1 shows the light-absorbing and optoacoustic properties of xPLORE-Apo-FL750© and xPLORE-CTRL-FL690© after injection in a custom-made tissue-mimicking agar phantom (at concentrations of 0.02 to 0.16 mg/mL). Figure 1a,b shows that the normalized optoacoustic spectra of both probes correlated well with the absorbance spectra obtained by spectrophotometry over the same spectral range (650–900 nm). The maximum absorbance value for xPLORE-Apo-FL750© was observed at around 740 nm in both the optoacoustic and spectrophotometric absorbance spectra. The maximum absorbance value for xPLORE-CTRL-FL690© was observed at 690 nm.

Figure 1c depicts the correlation between the probe concentration and light absorbance at 700 nm for each probe: an R^2^ value of 0.97 and 0.98 was observed for xPLORE-Apo-FL750© and xPLORE-CTRL-FL690©, respectively. We chose the 700 nm as the wavelength of reference because here we observed the highest OA signal intensity for both agents. Figure 1d shows the correlation between the probe concentration and measured optoacoustic signal at 700 nm for each probe: an R^2^ value of 0.98 and 0.96 was observed for xPLORE-Apo-FL750© and xPLORE-CTRL-FL690©, respectively. Furthermore, Figure 1e depicts a linear relationship between the measured light absorbance and optoacoustic signal for both probes, with an R^2^ value of 0.96 for both xPLORE-Apo-FL750© xPLORE-CTRL-FL690©. Finally, we tested the photostability of both probes by exposing them to pulsed laser light illumination at their maximum light absorbance wavelength (740 nm for xPLORE-Apo-FL750© and 690 nm for xPLORE-CTRL-FL690©) for 30 min. Figure 1f shows that after 20 min of illumination, only 30% of the initial optoacoustic signal was recorded. After 30 min, 15% of the xPLORE-Apo-FL750© signal and 5% of the xPLORE-CTRL-FL690© signal remained detectable.

### 3.1. xPLORE-Apo-FL750© as Optoacoustic Contrast Agent to Monitor Apoptosis

We next examined whether the apoptosis-targeting xPLORE-Apo-FL750© probe could allow tracking of chemotherapy-induced apoptosis in 4T1 tumors. Figure 2a illustrates the employed therapeutic scheme and MSOT imaging steps. Mice bearing 4T1 tumors were first scanned with MSOT to provide a baseline (Scan 1), then injected intravenously with the previously described (see Section 2) xPLORE-Apo-FL750© and xPLORE-CTRL-FL690© mixture and scanned again with MSOT 1 h after injection (Scan 2). Subsequently, mice were intraperitoneally treated with doxorubicin, a chemotherapeutic agent with a strong effect against a wide range of human malignant neoplasms [39]. A second dose of the abovementioned xPLORE mixture was injected intravenously 23 h after the doxorubicin injection. Mice were finally scanned again with MSOT 1 h later (Scan 3, 24 h after doxorubicin administration).

To explore the poly-caspase/tumor specificity of xPLORE-Apo-FL750©, animals were also injected with xPLORE-CTRL-FL690© and the results between the two agents were compared. Figure 2b,c,h,i (baseline measurements, Scan 1) show that virtually no optoacoustic signal from either probe was detectable in animals prior to administration of the xPLORE injection mixture. The presented greyscale optoacoustic images were acquired at 800 nm, the isosbestic point of hemoglobin (the point where the absorption of HbO_2_ and Hb are equal), a commonly employed wavelength for anatomical imaging with MSOT. However, 1 h after the first intravenous administration of the probe mixture (see Section 2), the apoptosis-specific probe xPLORE-Apo-FL750© demonstrated a relatively weak signal within the tumor region (Figure 2d,e), indicating the presence of low-level apoptosis in the absence of doxorubicin treatment (baseline tumor apoptosis). On the other hand, the control probe (Figure 2j,k) displayed virtually no signal within the tumor region following administration of the mixture and was confined to the vascular compartment at the tumor rim, demonstrating its absence of specificity for apoptotic tumor cells. Figure 2f,g (xPLORE-Apo-FL750©) and Figure 2l,m (xPLORE-CTRL-FL690©) show the unmixed MSOT imaging results for each probe 24 h after the administration of doxorubicin therapy and 1 h after re-administration of the xPLORE injection mixture. We observed that the xPLORE-Apo-FL750© signal intensity increased markedly in response to doxorubicin treatment (Figure 2f,g), consistent with the ability of doxorubicin to induce apoptosis. Quantification of the average xPLORE-Apo-FL750© signal amplitude in tumors showed that the probe-specific signal increased significantly from 0.25 ± 0.02 a.u. at baseline (Figure 2d,e, Scan 2) to 0.49 ± 0.05 a.u. (+96%, *p* < 0.0001) 24 h after doxorubicin treatment onset (Figure 2f,g, Scan 3). On the other hand, xPLORE-CTRL-FL690© remained confined to peripheral blood vessels following doxorubicin treatment (Figure 2l,m). As demonstrated in the provided optoacoustic images, the intravenous re-injection of the probe mixture at 23 h after the intraperitoneal doxorubicin administration resulted in a striking increase in xPLORE-Apo-FL750© signal intensity in the tumor core (Figure 2g, Scan 3), suggesting an enhanced apoptotic response to doxorubicin treatment.

### 3.2. MSOT Imaging of Intratumoral Apoptosis and Hypoxia In Vivo

**Figure 3:**
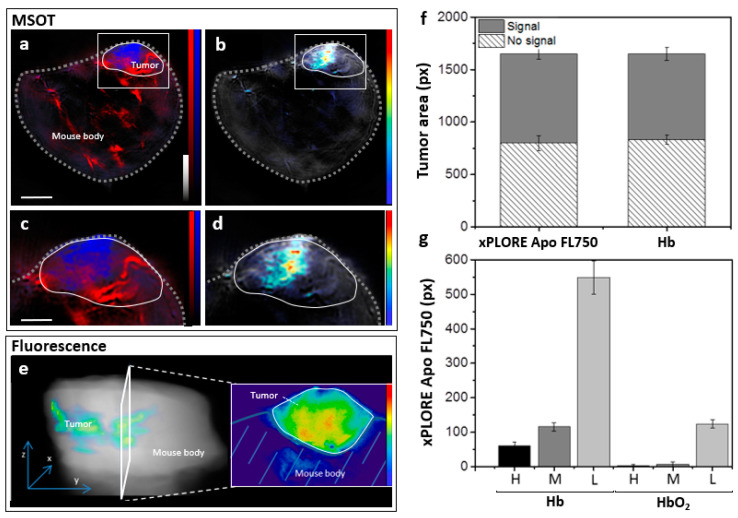
Intratumoral distribution of xPLORE-Apo-FL750©. Mammary gland 4T1 tumors were allografted into mice treated with doxorubicin for 24 h, then injected intravenously with xPLORE-Apo-FL750© and imaged first by MSOT in vivo and secondly by fluorescence imaging ex vivo. (**a**) Optoacoustic images showing overlaid deoxyhemoglobin (blue scale), oxyhemoglobin (red scale) distribution and anatomical background (800 nm: isosbestic point, greyscale). (**b**) Optoacoustic image showing the distribution of the xPLORE-Apo-FL750© probe (heat scale) merged with anatomical background (greyscale) in the same transverse plane as in (**a**). (**c**,**d**) Insets showing magnified views of the region enclosed in the white dotted lines in (**a**) and (**b**) respectively, indicating tumor margins. (**e**) Three-dimensional reconstruction of sequential planar ex vivo fluorescence images of tumor cryoslices (left) and exemplary two-dimensional transverse fluorescence image of a single tumor cryoslice (right). (**f**) Tumor area fractions containing xPLORE-Apo-FL750© or deoxyhemoglobin signal. (**g**) Spatial co-localization of xPLORE-Apo-FL750© and deoxyhemoglobin within the tumor tissue area after classifying pixels with positive xPLORE-Apo-FL750© signal into three signal intensity-based classes: high (H), medium (M) or low (L) signal.

In the next set of in vivo mouse experiments (*n* = 6), we employed MSOT to investigate the spatial overlap between the distribution of the apoptosis-specific probe and hypoxic tumor regions. We imaged the mice over the tumor region 24 h after doxorubicin injection and 1 h after the second intravenous injection of the probe. Figure 3a shows a transverse anatomical image of the mouse at the level of the tumor overlaid with blood oxygenation maps. The oxygenation maps showed a spatially varying pattern within the tumor; the core showed an intense signal of deoxygenated hemoglobin (Hb, blue), whereas the tumor rim displayed a relatively high content of oxygenated hemoglobin (HbO_2_, red) as clearly shown in the magnified tumor region (Inset, Figure 3c) Figure 3b shows the same anatomical image at 800 nm overlaid with the image illustrating the distribution of xPLORE-Apo-FL750©. As shown previously, the injected probe has a much higher accumulation in the tumor core compared to the surrounding tissues, which is also evident in the magnified inset (Figure 3d). Figure 3e shows the ex vivo fluorescence-based validation of the in vivo MSOT-extracted xPLORE-Apo-FL750© distribution. This ex vivo analysis showed that the fluorescence signal of the apoptotic probe accumulated at the tumor core, confirming the in vivo optoacoustic imaging findings.

These results suggest that xPLORE-Apo-FL750© accumulates predominantly in hypoxic regions within the tumor core. To further examine this hypothesis, we compared the intratumoral spatial overlap of the apoptosis-specific and Hb signals on a per-pixel basis. Figure 3f shows that approximately 50% of the tumor area pixels corresponded to xPLORE-Apo-FL750©- or Hb-signal, as calculated by segmentation analysis of the corresponding optoacoustic images. The effect size for the difference in the proportion of area with signal between xPLORE-Apo-FL750© and Hb is 0.404. Additionally, the 95% confidence intervals corresponding to Figure 3f are: xPLORE Apo FL750/No signal—(661.8, 936.2), Hb/No signal—832 ± 45 (743.8, 920.2), xPLORE Apo FL750/Signal: 849 ± 50 (751, 947), HB/Signal—816 ± 60 (698.4, 933.6). Furthermore, Figure 3g shows that approximately 95% of the pixels with medium or high xPLORE-Apo-FL750©-signal were located in areas assigned to Hb instead of HbO_2_. The same trend was observed for pixels with low xPLORE-Apo-FL750©-signal: approximately 80% of them belong to Hb-regions and only 20% to HbO_2_-regions. Indicatively, the effect size for the difference in areas with high signal (H) is 1.71. Furthermore, the 95% confidence intervals corresponding to Figure 3g are: Hb/H—(41.4, 80.6), Hb/M - (91.48, 138.52), Hb/L—(454.92, 643.08), HbO2/H—(−2.88, 8.88), HbO2/M—(−6.72, 20.72), HbO2/L—(100.48, 147.52). These observations showcase the capability of MSOT to image apoptosis and hypoxia simultaneously within the same tumor.

## 4. Discussion

Herein, we employ MSOT to simultaneously image different aspects of tumor pathophysiology based on injected dyes and endogenous contrasts. More specifically, we attempt to image both apoptotic responses to doxorubicin chemotherapy and hypoxia in mouse breast tumor allografts. Apoptosis detection is enabled by injecting the poly-caspase targeting probe xPLORE-Apo-FL750©, while tumor oxygenation is resolved by exploiting the different light-absorbing properties of HbO_2_ and Hb. Subsequent ex vivo fluorescence cryoimaging is also used to confirm the in vivo optoacoustic data.

As a form of programmed cell death, apoptosis plays a fundamental role in physiological processes ranging from the cellular (e.g., cellular turnover and embryonic development) to the systemic (e.g., nervous and the immune system) [40,41,42] and is a key factor in numerous diseases. Several preclinical studies have already demonstrated that MSOT can be used to image apoptosis, provided that the apoptosis-targeting injected probe absorbs light in the near-infrared (NIR) range (680–980 nm) [43,44,45,46,47].

Another key feature of solid tumors is the development of hypoxia, which similar to apoptosis, has been associated with resistance to various types of therapy, including chemotherapy and radiation treatment [48,49]. Furthermore, tumor hypoxia influences angiogenesis and leads to spatially irregular vascularity that may lead to changes at the cellular level and trigger pro-apoptotic stimuli [50,51,52]. Such stimuli exert selective stress on localized tumor microenvironments leading to even higher heterogeneity and enhancement of such processes within the tumor [53]. Consequently, imaging strategies that enable simultaneous detection of apoptosis and oxygenation in vivo could improve assessment of therapeutic efficacy in cancer patients and accelerate drug discovery and development [48,54].

In the first part of this work, we conduct an in vitro spectral characterization of the apoptosis-specific, poly-caspase-binding probe xPLORE-Apo-FL750© and the control probe xPLORE-CTRL-FL690©. Phantom experiments show that the normalized optoacoustic spectra of both probes strongly correlate with the absorbance spectra obtained by spectrophotometric analysis over the same spectral range (Figure 1a,b). Our in vitro experiments allow the accurate estimation of the optoacoustic absorption spectra for both probes at the NIR. While the two dyes display similar physical and chemical properties, they show different absorbance maxima. Thus, due to their unique absorption spectra, both probes can be identified and monitored/visualized separately, by means of spectral unmixing. In addition, photobleaching of the probes showed an approximately 20% drop in signal intensity after 10 min of continuous pulsed laser light illumination, while almost complete photobleaching of the probes was recorded after 30 min. Our data suggest that both probes are photostable, especially taking into account that the in vitro laser light exposure is much higher compared to the exposure expected in an in vivo experiment.

Next, to explore the specificity of xPLORE-Apo-FL750© for apoptosis, we co-inject xPLORE-Apo-FL750© and xPLORE-CTRL-FL690© in mice before and after intraperitoneal doxorubicin treatment and performed MSOT imaging at predetermined time intervals (Figure 2). First, following the injection of the probe mixture we observe a small but clear increase in xPLORE-Apo-FL750©-specific optoacoustic signal (Figure 2d,e), most probably suggesting the presence of low-level spontaneous apoptosis within the tumor, a phenomenon commonly observed over the course of solid tumor development [55]. On the contrary, the control probe shows virtually no signal at the tumor core (Figure 2j,k), demonstrating a lack of specificity for apoptotic tumor tissue. Secondly, we focus on evaluating the effect of doxorubicin treatment on tumor apoptosis by estimating the xPLORE-Apo-FL750©-specific optoacoustic signal 24 h after doxorubicin administration. Doxorubicin induces apoptosis by several molecular mechanisms, including DNA intercalation [56] and free radical formation [57]. Semi-quantitative analysis of intratumoral xPLORE-Apo-FL750© signal amplitude reveals that the probe-specific signal intensity following doxorubicin treatment is approximately double the signal recorded prior to treatment (see Section 3). On the contrary, the xPLORE-CTRL-FL690© which does not bind to caspases, is mainly retained within the blood stream throughout the mouse body and the tumor rim without penetrating into the tumor core (Scan 3, Figure 2j–m). Crucially, our in vivo MSOT imaging results are independently confirmed by ex vivo fluorescence cryoimaging analysis [38], which showed a preferential accumulation of the xPLORE-Apo-FL750© probe at the cores of tumors and a similar overall spatial distribution of the probe to the one revealed by MSOT.

Finally, we investigated the relation between the spatial distributions of the apoptosis-specific xPLORE-Apo-FL750© probe and Hb/HbO_2_ (oxygenation markers) in 4T1 tumors, following doxorubicin treatment (Figure 3). MSOT provided detailed maps (spatial resolution of ≈150 μm) of both tumor Hb/HbO_2_ content and xPLORE-Apo-FL750©-distribution not only within the tumor but also throughout the whole-body mouse thickness. This unique capability of MSOT enabled quantitative evaluation of the relationship between doxorubicin-induced apoptosis (based on xPLORE-Apo-FL750©) and hypoxia (based on Hb) within the tumor mass. Co-localization analysis of Hb and probe-specific optoacoustic signals showed that hypoxia-related Hb pixels were present within approximately half of the tumor mass (Figure 3f) and highly overlapped with the pixels corresponding to xPLORE-Apo-FL750© (Figure 3g). Altogether, our findings demonstrate the capability of MSOT to provide multi-aspect imaging of tumor pathophysiology (e.g., oxygenation/hypoxia and apoptosis) based on simultaneous and co-registered imaging of both injected dyes (e.g., xPLORE-Apo-FL750©) and endogenous tissue chromophores (e.g., Hb/HbO_2_).

The current study does not come without limitations. Regarding the in vitro studies on the injected probes: The number of the calculated and presented points (*n* = 4) is relatively low and this could limit the generalization/interpretability of our results. Furthermore, the small sample size, even if it provides useful insights into the investigated phenomena, could be radically expanded in future studies. As general limitations of the MSOT technology, we would like to discuss the low penetration depth (approximately 2–4 cm depending on tissue type), which might be sufficient for small animal imaging but limits the use of the technique in selected translational and clinical applications. Furthermore, the per-pixel spectral unmixing method applied could also introduce calculation errors due to motion artifacts.

In summary, in the current study MSOT was successfully used to simultaneously visualize apoptosis and tissue oxygenation during doxorubicin chemotherapy in breast cancer tumors in vivo. Our experimental findings demonstrate the usability of xPLORE-Apo-FL750© as a potential small-molecular contrast agent for preclinical optoacoustic imaging of apoptosis and reveal novel insights into its intratumoral distribution in correlation to tumor oxygenation/hypoxia. On one hand, accurate apoptosis monitoring would facilitate its discrimination from other cell death processes (e.g., necrosis) and the conduction of longitudinal studies on the efficacy of novel apoptosis-targeting schemes. On the other hand, simultaneous exploration of different biological processes, such as apoptosis and hypoxia, with a single test may boost progress towards an integrated understanding of tumor pathophysiology in dedicated animal models with high convenience and lower cost. Furthermore, recent advances in optoacoustic imaging for preclinical and clinical use [17,26,27] offer great versatility for the conduction of translational studies. Thus, our results provide useful insights into the assessment of chemotherapy-induced apoptosis deep in tumors in vivo, as well as the connection between apoptosis and tumor hypoxia in breast tumors and suggest MSOT as a non-invasive tool for the simultaneous and multi-aspect exploration of complex pathophysiological processes such as those in breast cancer.

## Data Availability

The data presented in this study are available upon request from the corresponding author. The data are not publicly available due to privacy/internal regulations.
